# A University Diploma in Tropical Medicine

**Published:** 1904-02-27

**Authors:** 


### A University Diploma in Tropical Medicine.

The authorities of the University of Cambridge have decided to institute a Diploma in Tropical Medicine and Hygiene. The diploma will be conferred after an examination conducted by the University State Medicine Syndicate, the first examination to take place in August of the present year. The study of tropical medicine has in recent years been marked by such brilliant and startling results, and its practical importance both to science and humanity has received such impressive testimony, that there will be no difficulty in allowing the fitness of its academic recognition. And it is entirely in harmony with recent traditions that the University of Cambridge should afford this recognition. Whatever may be said of the essential identity of pathological processes in all climates and latitudes, it remains true that in actual practice disease in the tropics is marked by special features and demands special training and study. The important discoveries of the last few years have emphasised this position and have forced the conclusion that for those who aspire to positions of medical responsibility in the more remote parts of the Empire an addition to the ordinary curriculum is essential. For this, admirable provision has already been made in the schools of tropical medicine at London and Liverpool, and no one will grudge to those who submit to the discipline there offered the prospect of a University distinction and diploma. It may reasonably be expected that such a prospect will have a beneficial effect both on the individual worker and on the development of the subjects included within the scope of the examination. The existing schools do already grant to their students a certificate of efficiency, the value of which is officially recognised. But it will be a decided gain to have a diploma which is guaranteed by a leading British University and is conferred only after a defined course of study and a comprehensive and impartial examination. So far from instituting a rivalry to the existing schools, the new development is likely to increase their range and usefulness. It may be expected both to multiply their students and also to extend the period during which each individual student will desire to prosecute his studies. In this regard it is to be noted that the candidate for the diploma must produce evidence that he has studied at a hospital for tropical diseases, and also that the clinical part of the examination will be conducted at such a hospital, where cases will be submitted for diagnosis and comment. These conditions are to be highly commended, and suggest, what certainly ought to be the case, that the University authorities recognise fully the position attained by the London and Liverpool schools and desire to associate those interested in these schools with their present proposals. Moreover, the profession would certainly not look with favour on any scheme which attempted to replace clinical and laboratory training by mere theoretical knowledge as the compulsory avenue to academic recognition. The object and motive of the Cambridge proposal, as we understand it, is to provide a chance for such recognition to those who have diligently pursued the opportunities offered by the existing schools of tropical medicine. This, we feel sure, will command general respect, and may be confidently expected to secure the support of those whose unfailing courage and brilliant and persevering work have placed the study of tropical medicine in this country in the worthy position it now fortunately occupies. The range of the new examination is necessarily wide, but it seems to us both sound and judicious. In addition to specific knowledge of the various forms of disease likely to be met with in tropical countries, it includes parasitology, the methods of pathological and bacteriological investigation, and hygiene and methods of sanitation applicable to such climates. There is no regulation determining the length of time that these special studies shall be pursued. All that is required is that the candidate shall have held a registrable qualification for not less than 12 months, and that he produce evidence of study at a hospital for tropical diseases. For the present this may suffice, and it is reasonable to consider the position of those who, for longer or shorter periods, have been practising abroad, and who during a short stay at home may desire to secure the University diploma. But, as in the parallel instance of the public health diploma, it is probable that sooner or later the authorities will demand a definite curriculum of clinical and laboratory work, and in our judgment this will have to come early rather than late. The Home and Colonial Governments, as well as private individuals, have a large interest in the efficiency of those who practice medicine in British possessions, and they may be expected to encourage all measures to promote so desirable an end. The Cambridge scheme favours this result, and we regard it both as good in itself and also as having the promise of even better things in the not remote future.

